# Allosteric coupling between G‐protein binding and extracellular ligand binding sites in GPR52 revealed by ^19^F‐NMR and cryo‐electron microscopy

**DOI:** 10.1002/mco2.260

**Published:** 2023-04-10

**Authors:** Yanliu Fan, Xi Lin, Benxun Pan, Bo Chen, Dongsheng Liu, Kurt Wüthrich, Fei Xu

**Affiliations:** ^1^ iHuman Institute School of Life Science and Technology Shanghai Key Laboratory of High‐resolution Electron Microscopy ShanghaiTech University Shanghai China; ^2^ Department of Integrative and Computational Biology Scripps Research La Jolla California USA; ^3^ Department of Biology ETH Zürich Zürich Switzerland

Dear Editor,

GPR52 is a class‐A orphan GPCR, which has been reported as a target for treating Huntington's disease, cognitive impairment, schizophrenia, and neuromuscular disorders. Previous structure determinations showed that GPR52 adopts an active conformation when bound to G‐protein in the absence of any ligand.^1^ A self‐activation model was proposed, attributed to a built‐in agonist motif embedded in the extracellular loop 2 (ECL2) near the orthosteric ligand binding pocket; this would also explain the high basal activity of GPR52. The synthetic ligand c17 is bound to a GPR52 side pocket, as shown in the co‐crystal structure.^1^ Recently, some new molecules with a similar chemical structure to c17 have entered pre‐clinical trials for GPR52‐related disorders.^2^

Structural and conformational dynamics studies of GPCRs are crucial for the screening of new ligands and understanding the molecular mechanisms of previously untapped drug targets. Here, we show a new cryo‐electron microscopy (cryo‐EM) structure of GPR52 bound with c17 at the extracellular side and G‐protein at the intracellular side. ^19^F‐NMR studies^3^ of the complex by observation of the trifluoromethyl group in c17 (Figure 1A) revealed local polymorphisms in GPR52. ^19^F‐NMR data also indicate localized differences in the extracellular ligand binding pocket between the GPR52–c17 complex and the ternary complex with the G‐protein.

**FIGURE 1:**
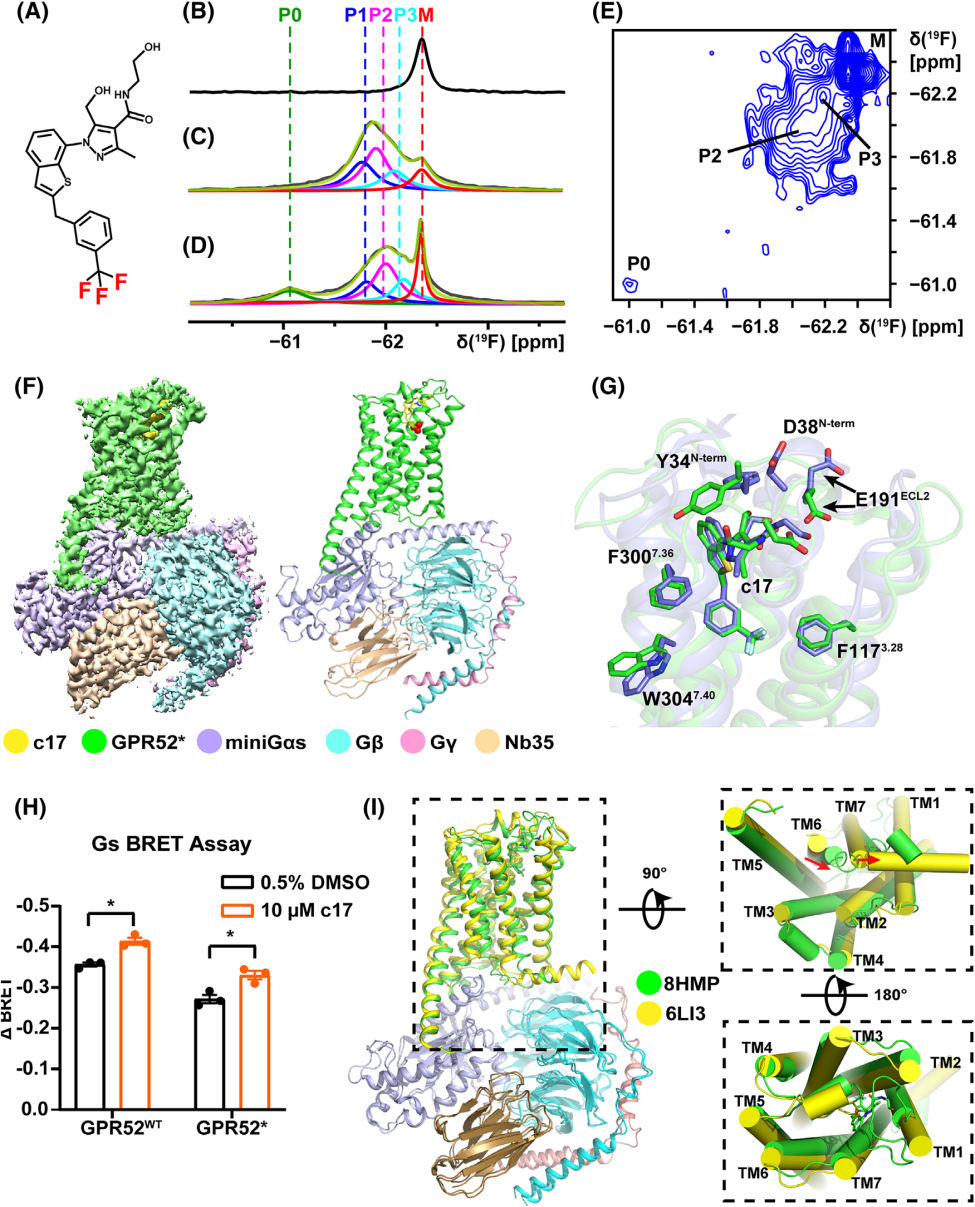
Conformational ensembles of GPR52 in complex with G‐protein studied by ^19^F‐NMR and cryo‐electron microscopy (cryo‐EM). (A) Chemical structure of the ligand c17 with the trifluoromethyl group highlighted in red. (B) ^19^F‐NMR signal of c17 in lauryl maltose neopentyl glycol (LMNG)/cholesteryl hemisuccinate tris salt (CHS) mixed micelles at 298 K. (C, D) 1D ^19^F‐NMR spectra of the GPR52*–c17 and GPR52*–c17–G‐protein complexes in mixed micelles of LMNG and CHS at 298 K. The black lines represent the experimental ^19^F‐NMR signals of the –CF3 group. Lorentzian deconvolution showed that this envelope contained three peaks P1, P2, and P3 (blue, pink, and cyan). The peak P0 (green) is seen only for the ternary complex of c17, GPR52*, and G‐protein. M (red) represents micelle‐associated “free” c17; the sum of the components is shown in green‐yellow. (E) 2D [^19^F,^19^F]‐EXSY spectrum of the GPR52*–c17–G‐protein complex collected at 298 K with a mixing time of 400 ms. The diagonal peak positions of P0, P2, P3, and M are labeled. (F) Overall cryo‐EM density map and atomic model of GPR52*–c17–G‐protein complex. GPR52*, green; miniGαs, purple; Gβ, cyan; Gγ, pink; Nb35, wheat. (G) Comparison of ligand c17 binding pocket in GPR52^crystal^–c17 and GPR52*–c17–G‐protein complex structures (PDB: 6LI0 and 8HMP, blue and green), c17 is shown in yellow stick presentation, the residues (D38^N‐term^, F117^3.28^, E191^ECL2^, F300^7.36^, and W304^7.40^) within 4 Å of the ligand are shown as sticks. (H) The activation of GPR52^WT^ and GPR52* in the absence and presence of the ligand c17 were monitored by bioluminescence resonance energy transfer (BRET) assay, ΔBRET: the change of bioluminescence resonance energy transfer value. Significance was determined by two‐way analysis of variance (ANOVA) without repeated measures, followed by Dunnett's post hoc test (*p < 0.05). Data are mean ± s.e.m. (*n* = 3). (I) Superposition of GPR52 structures, GPR52*–G‐protein (PDB: 6LI3, yellow) and GPR52*–c17–G‐protein (PDB: 8HMP, green), viewed from the intracellular and extracellular sides (bottom and top view). The movements of the intracellular portion of TM6 and TM7 were indicated by red arrows.

To investigate the dynamics of the ligand binding pocket of GPR52, the near‐wild type receptor and some variants were expressed in *Sf*9 insect cells (Figure S1). The –CF_3_ group of c17 (Figure 1A) serves here as a natural ^19^F‐NMR probe for studies of the complex with GPR52. For NMR measurements, the GPR52 complexes were reconstituted in lauryl maltose neopentyl glycol/cholesteryl hemisuccinate tris salt mixed micelles.

1D ^19^F‐NMR spectra of the ligand c17 in the free state as well as bound to GPR52* (see Table S1 for the construct information) and in the tertiary complex with the G‐protein bound were collected. The ^19^F‐NMR signal at −62.3 ppm (M in Figure 1B) was assigned to the trifluoromethyl group of the free ligand c17 in micelles by comparing the spectra in Figure 1B–D. Broad peaks at the lower field were assigned to c17 bound to GPR52*, based on ring current shift calculations (Table S2).^4^ Lorentzian deconvolution was used to identify overlapped subpeaks. The resonance of c17 bound to GPR52* contains three components P1, P2, and P3 at −61.8, −62.0, and −62.1 ppm (Figure 1C and Table S3), representing three substates. Addition of G‐protein to the GPR52*–c17 complex revealed a new peak, P0, centered at −61.1 ppm (Figure 1D and Table S3). The observation of the ^19^F‐NMR signal of bound c17 thus showed that bound G‐protein has a long‐range impact on its extracellular ligand binding pocket.

The impact of various mutations on the polymorphisms of GPR52 was studied by observation of the ^19^F‐NMR signal of c17 with GPR52^crystal^, GPR52^WT^, GPR52^A130W^, and GPR52^C314P^ (Figure S2A–D and Table S1). Lorentzian deconvolution showed similar chemical shift distributions for all these constructs. P0 appeared consistently with G‐protein bound (Figure S2E and Table S4).

The presence of the separate peaks P0, P1, P2, and P3 shows that the exchange rate between these conformational substates is slow on the chemical shift time scale. To further explore possible differences between the conformational exchange rates in different variants, 2D [^19^F,^19^F]‐EXSY experiments were conducted (Figure 1E and Figure S2F–H). The presence of cross‐peaks between P1, P2, or P3 in the GPR52–c17 complex shows that the exchange rates between these conformational states are within the millisecond range (Figure 1E and Figure S2F–H). The absence of cross peaks with P0 indicates that the exchange between P0 and the other states is too slow to be seen with the current measurements, with *k*_ex_ ≤ 10 s^−1^.

That c17 is similarly bound to GPR52^WT^ and GPR52* was confirmed with bioluminescence resonance energy transfer (BRET) assays, to measure G‐protein dissociation and observe changes of the BRET signal induced by c17 in both cases (Figure 1H).

To determine the structure of GPR52 in the c17‐bound G‐protein coupled state, we assembled a GPR52–G‐protein complex in the presence of c17. The final complex contained the purified GPR52* protein, c17, miniGαs, Gβ1γ2, and the Nb35 (Figure S3). We determined this complex structure by single‐particle cryo‐EM at a global resolution of 2.7 Å (Figure 1F, Figure s3– 6, and Table S5). Most residues are well‐defined in all protein components due to the high resolution (Figure S5). Overall, the ligand pocket revealed in this cryo‐EM structure is of sufficiently high resolution to enable the following analysis (Figure S5). Although the receptor part is consistent with previously published GPR52 structures (PDB: 6LI0, 6LI1, 6LI2, and 6LI3), the c17 ligand pocket showed notable differences when compared to the crystal structure of the GPR52–c17 complex (PDB: 6LI0), which can be attributed to the presence of the G‐protein (Figure 1G). The major difference is located in the N‐terminal region and the extracellular loops, including a rotation of the side chains of two residues D38^N‐term^, Y34^N‐term^, and a new contact of c17 with E191^ECL2^ in the new structure. Notably, the whole binding pocket for c17 was expanded when the receptor coupled with G‐protein, which was mainly orchestrated by motions of the side chains of aromatic residues. F117^3.24^ showed an inward movement of 1 Å along the aromatic plane, F300^7.36^ underwent a clockwise rotation of 20°, and W304^7.40^ had a rotation of 30°. The resulting overall volume change of the ligand binding pocket was approximately 300 Å^3^ (Figure S7).

It is intriguing that the structure of the GPR52*–c17–G‐protein complex showed a unique conformation at the intracellular side, which is contradictory to all other observed GPCR cases. In comparison to the structure of the GPR52*–G‐protein complex in the absence of c17 (PDB: 6LI3), we observed an “inward” movement of the intracellular portion of TM6 and an “outward” motion of the intracellular part of TM7 (Figure 1I). Residue R139^3.50^ is in close contact with D138^3.49^, which is in sharp contrast to other representative class‐A GPCRs (CB1R, NK1R, D2R, and A_2A_R) in active states when bound to G‐protein, where the “ionic lock” was broken (Figure S8). Whether such a “hybrid” conformation of GPR52 represents an additional, new step in the activation mechanism may deserve further investigation.

The present integrated use of solution ^19^F‐NMR and cryo‐EM revealed the long‐range impact of the bound G‐protein on an extracellular GPR52 ligand pocket, as well as conformational polymorphisms of the extracellular binding pocket for the ligand c17 seen in the ^19^F‐NMR signal of c17. The low‐field peak P0 in the G‐protein‐bound state (Figure 1D) showed that the bound G‐protein allosterically changed the ligand binding pocket in a small population of GPR52 molecules revealing long‐range cross‐talk between ligand and G‐protein (Figure S9).

The combination of ^19^F‐NMR and cryo‐EM provides us with high‐resolution structures as well as dynamics and interaction information. The present work illustrates that in addition to ^19^F‐NMR probes attached to the GPCR^3^ or to orthosteric ligands,^5^ information on functional‐related long‐range interactions can also be obtained by observation of bound allosteric modulators. The information thus obtained shall provide insight into allosteric modulation mechanisms and functional ligand discovery for GPR52 to accelerate current medicinal chemistry efforts on this emerging drug target. We trust that this study will inspire future investigations on other orphan GPCRs by using combined biophysical approaches, in our laboratories as well as elsewhere.

## AUTHOR CONTRIBUTIONS

Y.F. conducted cloning, protein purification, NMR sample preparation, NMR data collection, and analysis; X.L. performed GPR52*‐c17‐G‐protein purification, cryo‐EM sample preparation, cryo‐EM data collection, and structural analysis; B.P. performed the NMR experiments; B.C. performed cryo‐EM data processing, model building, and refinement; F.X., D.L., and K.W. supervised the experiments. Y.F., X.L., B.P., D.L., K.W., and F.X. wrote the manuscript with input from all authors. All authors have read and approved the final manuscript.

## CONFLICT OF INTEREST STATEMENT

The authors declare no conflict of interest.

## FUNDING INFORMATION

The Ministry of Science and Technology of China (grant number 2018YFA0507000 to Fei Xu), Shanghai Science and Technology Plan (grant number 21DZ2260400 to Fei Xu), the National Natural Science Foundation of China (grant number 31971153 to Dongsheng Liu), and the China Postdoctoral Science Foundation (grant number 2021M693166 to Xi Lin).

## ETHICS STATEMENT

Not applicable.

## Supporting information



Supporting Information

## Data Availability

The cryo‐EM map and coordinates for GPR52*–c17–G‐protein structure reported in this study have been deposited in EMDB and PDB with accession numbers of EMD‐34902 and 8HMP, respectively.
