# Supplementary material for: Allosteric coupling between G‐protein binding and extracellular ligand binding sites in GPR52 revealed by 19F‐NMR and cryo‐electron microscopy
Source: MedComm (2020). 2023 Apr 10;4(2):e260. doi: 10.1002/mco2.260 (PMC10085714; doi:10.1002/mco2.260)
Supplement: Supplementary file 1 — Supporting Information [file MCO2-4-e260-s001.docx]

Supplemental Materials

**Allosteric coupling between G-protein binding and extracellular ligand binding sites in GPR52 revealed by ^19^F-NMR and cryo-EM**

Yanliu Fan^1,#^, Xi Lin^1,#^, Benxun Pan^1^, Bo Chen^1^, Dongsheng Liu^1,*^, Kurt Wüthrich^1,2,3,*^, Fei Xu^1*^

^1^iHuman Institute, School of Life Science and Technology, Shanghai Key Laboratory of High-resolution Electron Microscopy, ShanghaiTech University, Shanghai, China.

^2^Department of Integrative and Computational Biology, Scripps Research, La Jolla, CA, USA.

^3^Biology Department, ETH Zürich, Switzerland.

^#^Yanliu Fan and Xi Lin contributed equally to this work.

*Correspondence Fei Xu, Kurt Wüthrich and Dongsheng Liu.

**This file includes:**

Methods and Materials

References

Figures S1 to S9

Tables S1 to S5

**Methods and Materials**

**Protein expression and purification of GPR52 and Gβ1γ2**

The human GPR52 gene was subcloned into the expression vector pFastBac1. The construct contained residues 1–340 of GPR52 and 3C protease cleavage site which was used to remove the 10×His-Tag at C terminus. Haemagglutinin (HA) signal peptide, Flag tag and b562RIL (BRIL)^1, 2^ were added to the N terminus to improve protein yield and for detection which were then removed by TEV enzyme. To improve protein stability, two mutations A130^3.14^W, C314^7.50^P were introduced. The protein of GPR52[1–340, A130W, C314P] was named GPR52*, and the protein of GPR52 used for previously crystal structure determination was named GPR52^crystal^. Gβ1γ2 was cloned to pFastBac Dual vector. We used the Bac-to-Bac Baculovirus System (Invitrogen) in *Spodoptera frugiperda* (*Sf9*) cells for the expression of GPR52 and Gβ1γ2. These cells were infected at a density of 2 × 10^6^ cells per mL with baculovirus. Cells were grown at 27 °C and collected at 48 h after infection, and cell pellets were stored at −80 °C for future use.

The cell pellets of GPR52 protein were thawed and washed with a low-salt buffer (10 mM HEPES pH 7.5, 20 mM KCl, 10 mM MgCl_2_, protease inhibitor cocktail (Roche)). This was followed by three washes with a high-salt buffer (10 mM HEPES pH 7.5, 1 M NaCl, 20 mM KCl, 10 mM MgCl_2_, protease inhibitor cocktail). Before solubilization, purified membranes were incubated with 20 μM c17 and 2 mg/mL iodoacetamide (Sigma) at 4 °C for 1 h. GPR52 was extracted from the membrane by adding HEPES, NaCl, lauryl maltose neopentyl glycol (LMNG, Anatrace), and cholesteryl hemisuccinate (CHS, Sigma) to the membrane solution to a final concentration of 100 mM, 500 mM, 1.0% (w/v) and 0.2% (w/v), respectively, and stirred for 2 h at 4 °C. The supernatant was collected by centrifugation at 35,000 rpm for 30 min and incubated with TALON IMAC resin (Clontech) and 20 mM imidazole at 4 °C overnight. Then the resin was washed with 15 column volumes (CVs) of buffer (50 mM HEPES pH 7.5, 500 mM NaCl, 5% (v/v) glycerol, 0.05% (w/v) LMNG, 0.01% (w/v) CHS, 10 mM MgCl_2_, 20 mM imidazole, 20 µM c17), followed by 15 CVs of wash buffer (25 mM HEPES pH 7.5, 100 mM NaCl, 5% (v/v) glycerol, 0.03% (w/v) LMNG, 0.006% (w/v) CHS, 40 mM imidazole, 20 µM c17). Finally, the protein was eluted using 3 CVs of elution buffer (25 mM HEPES pH 7.5, 100 mM NaCl, 5% (v/v) glycerol, 0.01% (w/v) LMNG, 0.0002% (w/v) CHS, 220 mM imidazole, 20 µM c17). The protein was concentrated to 1-3 mg/mL for future use. For NMR observation of the receptor-bound c17, no additional c17 was added after membrane solubilization.

For purification of heterodimeric Gβ1γ2 protein,^3^ the genes of Gβ1 and Gγ2 (human) were cloned into pFastBac-Dual vector and expressed in *Sf9* for 48 h. The cell pellets from 2 L of Gβ1γ2 were thawed and resuspended to 50 mL in buffer (30 mM Tris pH 8.0, 100 mM NaCl, 5 mM MgCl_2_, 5 mM imidazole, protease inhibitor cocktail, 50 μg/mL DNase I, 100 μM DTT). Cells were broken by sonication and clarified by centrifugation (38,000 g for 1 h). The supernatant was loaded onto 2 mL Ni^2+^ affinity chromatography. The column was washed with 20 mL of buffer (20 mM Tris pH 8.0, 300 mM NaCl, 30 mM imidazole, 10% glycerol, 1 mM MgCl_2_). The column was eluted with 6 mL buffer (20 mM Tris pH 9.0, 50 mM NaCl, 500 mM imidazole, 10% glycerol, 1 mM MgCl_2_). The elute was diluted to 60 mL in buffer (20 mM Tris pH 9.0, 50 mM NaCl, 10% glycerol, 1 mM MgCl_2_, 1 mM DTT) and loaded onto a 5 mL HiTrap Q FF column (GE Healthcare) at 5 mL/min. The Q FF column was washed with 40 mL buffer (20 mM Tris pH 9.0, 50 mM NaCl, 10% glycerol, 1 mM MgCl_2_, 1 mM DTT) and eluted with a linear gradient of 50–300 mM NaCl in buffer (20 mM Tris pH 9.0, 50 mM NaCl, 10% glycerol, 1 mM MgCl_2_, 1 mM DTT). The protein solution was concentrated to a volume of 1 mL and loaded onto a Superdex 200 10/300 column (GE Healthcare) in buffer (10 mM HEPES pH 7.5, 100 mM NaCl, 10% glycerol, 1 mM MgCl_2_, 0.1 mM TCEP). Peak fractions of heterodimeric Gβ1γ2 protein were concentrated to 5 mg/mL for future use.

**Expression and purification of miniGαs399 and Nb35**

The miniGαs399 and Nb35 were expressed in *Escherichia Coli* (*E. coli*) system. Gαs subunit of miniGαs (mGαs) used in this study was the same as that was used in a previous study of the cryo-EM structures of A_2A_AR–mini-Gs–Nb35 and GPR21-mini-Gs-Nb35.^4-6^ In Brief, the miniGαs399 was expressed in *E. coli* strain BL21 (DE3) and cultured in TB media. Induction of expression was achieved by adding 50 μM of IPTG when OD600 reached 0.6. Cells were grown at 25 °C and collected after 20 h, and cell pellets were stored at −80 °C for future use. MiniGαs protein was purified by Ni^2+^ affinity chromatography. The protein solution was concentrated to a volume of 5 mL and loaded onto a Superdex 200 10/600 column (GE Healthcare). Peak fractions of miniGαs protein were concentrated to 20 mg/mL for future use.

The purification of Nb35 was performed following previous protocols.^4, 7^ In Brief, The Nb35 was cloned in pET22b vector and expressed in *E. coli* strain BL21 (DE3) and cultured in TB media supplemented with glucose (0.2%) and MgSO_4_ (5 mM). Cultures were grown at 30 °C until OD600 reached 0.8. Expression was induced with 25 μM IPTG and the temperature reduced to 25 °C. Cells were harvested 20 h post-induction by centrifugation at 4,000 g for 20 min. The pellet was resuspended from 1 L of *E. coli* culture in buffer (20 mM HEPES pH 7.5, 100 mM NaCl, 10 mM imidazole, 5 mM MgCl_2_, protease inhibitor cocktail, 50 μg/mL DNase I, 100 μg/mL lysozyme) and lysed by sonication. After another centrifugation (40,000 rpm, 30 min), the supernatant was loaded onto 2 mL Ni^2+^ affinity chromatography. The column was washed with 20 mL buffer (20 mM HEPES pH 7.5, 500 mM NaCl, 40 mM imidazole). The column was eluted with 6 mL buffer (20 mM HEPES pH 7.5, 100 mM NaCl, 500 mM imidazole). The protein solution was concentrated to a volume of 1 mL and loaded onto a Superdex 200 10/300 column (GE Healthcare) in buffer (10 mM HEPES pH 7.5, 100 mM NaCl, 10% glycerol). Peak fractions of Nb35 protein were concentrated to 20 mg/mL for future use.

**Purification and formation of** **GPR52*****–c17–G-protein complex**

Purified GPR52* receptor, heterodimeric Gβ1γ2, miniGαs, and Nb35 were mixed in a 1:1.2:1.5:2 ratio followed by the addition of apyrase (1 unit). The mixture was incubated at 4 °C overnight. The GPR52*–c17–G-protein complex was loaded on Superdex 200 10/300 column (GE Healthcare) with SEC buffer (20 mM HEPES pH 7.5, 100 mM NaCl, 0.00075% (w/v) LMNG, 0.00025% glycol-diosgenin (GDN), 0.00025% (w/v) CHS, 100 µM DTT, 5 μM c17). Peak fractions containing GPR52*–c17–G-protein complex were concentrated to 2.5 mg/mL for future studies.

**Cryo-EM sample preparation**

3 μL of the purified GPR52*–c17–G-protein complex was applied to glow-discharged 400-mesh Au grids (Quantifoil, R1.2/1.3). Excess sample was removed by blotting with filter paper for 3.5 s before plunge-freezing in liquid ethane using a FEI Vitrobot Mark IV at 100% humidity and 8 °C.

**Cryo-EM image acquisition**

All the datasets were collected on a Titan Krios 300 kV electron microscope (Thermo Fisher Scientifics) equipped with a GIF Quantum energy filter (20 eV energy slit width, Gatan), and a K3 Summit direct electron detector (Gatan) at 105,000 nominal magnification (calibrated pixel size: 0.832 Å/pixel) and 15 e^-^/pixel^2^/s. The movies were recorded using the super resolution counting mode by SerialEM^8^ which applies the beam image shift acquisition method with one image near the edge of each hole and saved as non-gain normalized TIFF files. A 50 µm C2 aperture was always inserted during the data collection period. The defocus ranged from -0.7 to -2.2 µm. For each movie stack, a total of 40 frames were recorded, yielding a total dose of 60e^-^/Å^2^. A total of 1999 movies were recorded.

**Cryo-EM image processing**

The dataset was motion corrected with MotionCor2^9^ and no frame grouping. Both the dose weighted and non-dose weighted averages were saved, and the CTF parameters were estimated based on the non-dose-weighted averages using CTFFind.^10^ Only images with the highest resolution of less than 4 Å were selected for further processing. Moreover, images with empty holes, visible contamination or large carbon regions by manual examination were also removed. A total of 1,668 movies were finally chosen for particle picking. To avoid potential bias about the structural conformation in the dataset, a Laplacian-of-Gaussian blob picker in Relion 3.1 was first applied to pick particles. 2D class averages with diverse orientations and clear secondary features were selected as the 2D templates for another round of autopicking process by Relion 3.1, yielding an initial particle stack of 1,176,096 particles. Further rounds of 2D classification were applied to eliminate particles without visible secondary features by Relion 3.1,^11^ yielding a dataset containing 514,897 particles in total. A 40 Å low-pass filtered GPR52*–c17–G-protein complex structure (PDB: 6LI3) was selected as the initial model for further processing.

Subsequent 3D refinements were all performed by Relion 3.1. For the first round 3D classification, the 40 Å low-pass filtered GPR52*–c17–G-protein complex structure was also used to divide the dataset into 3 different 3D classes. 390,563 particles which were associated with 3D maps with GPCR complex features were grouped together and were subjective for further heterogeneous refinement into 3 different classes. Finally, a total of 294,219 particles were selected for homogeneous refinement and post-processing, yielding a map with resolution of 3.13 Å determined by gold standard resolution test (cutoff: FSC=0.143). Then, the dataset was subjected to further rounds of CTF refinement and Bayesian polishing and post-processing by Relion 3.1, and the final resolution was improved to 2.77 Å with a soft-edge mask based on the 3D refined map with a 6 pixel extension and a 6 pixel soft edge. The local resolution was estimated using the cryosparc v2.15 “local resolution estimation” function (0.143 cut-off).

**Bioluminescence resonance energy transfer (BRET) assay**

Gs probes were generated according to previous report.^12^ The G protein dissociation assay was performed as previously described.^13^ Briefly, HEK293 cells were transiently co-transfected with varying amounts of plasmids encoding GPR52^WT^ or GPR52* together with Gs BRET probes. 24 hours after transfection, cells were distributed into a 96-well microplate and incubated for additional 24 hours at 37 °C. For BRET signal measurement, the transfected cells were incubated with 60 μL HBSS for 5 min, cells were then treated with 30 μL of ligand c17 for 5 min, the BRET signal finally was directly recorded using a Mithras LB940 microplate reader (Berthold Technologies) after the addition of 10 μL of freshly prepared 50 μM coelenterazine 400a (Nanolight Technologies). The BRET signal was calculated as the ratio of light emission at 510 nm and light emission at 400 nm.

**NMR spectroscopy**

^19^F-NMR spectra were collected on a Bruker AVANCE III HD 600 MHz spectrometer equipped with a TCI ^1^H/^19^F-^13^C-^15^N triple resonance cryoprobe with shielded z-gradient coil. The ^19^F-chemical shifts were referenced using an internal standard TFA at −75.5 ppm. The 1D ^19^F-NMR experiments were recorded with a data size of 4,096 complex points and an acquisition time of 72 ms, and 8,192 scans at 298 K per experiments. 2D [^19^F,^19^F]-EXSY experiments were recorded with a data size of 1,024 and 40 complex points in the direct and indirect dimensions, respectively; 1,024 scans were accumulated per increment. The line broadening factor was 30 Hz in ^19^F-NMR spectra. The data were analyzed by using Bruker Topspin 4.1.4 and MestReNova 14.

**The calculation of ligand binding pocket volume**

To compare the c17 binding pocket volume in GPR52^crystal^–c17 and GPR52*–c17–G-protein, Pocket Cavity Search Application^14, 15^ (POCASA, an automatic ligand-binding-site prediction program) was applied. Probe radius was set to 2 Å, the size of unit grid was 1 Å and the result was shown in Supplementary information, Figure S7.

**References**

1. Chun E, Thompson AA, Liu W, et al. Fusion partner toolchest for the stabilization and crystallization of G protein-coupled receptors. *Structure*. 2012;20(6):967-76.

2. Zhang K, Wu H, Hoppe N, Manglik A, Cheng Y. Fusion protein strategies for cryo-EM study of G protein-coupled receptors. *Nat Commun*. 2022;13(1):4366.

3. Carpenter B, Tate CG. Expression and Purification of Mini G Proteins from Escherichia coli. *Bio Protoc*. 2017;7(8)

4. Garcia-Nafria J, Lee Y, Bai X, Carpenter B, Tate CG. Cryo-EM structure of the adenosine A(2A) receptor coupled to an engineered heterotrimeric G protein. *Elife*. 2018;7:e35946.

5. Lin X, Chen B, Wu Y, et al. Cryo-EM structures of orphan GPR21 signaling complexes. *Nat Commun*. 2023;14(1):216.

6. Carpenter B, Nehmé R, Warne T, Leslie AG, Tate CG. Structure of the adenosine A(2A) receptor bound to an engineered G protein. *Nature*. 2016;536(7614):104-7.

7. Carpenter B, Tate CG. Engineering a minimal G protein to facilitate crystallisation of G protein-coupled receptors in their active conformation. *Protein Eng Des Sel*. 2016;29(12):583-594.

8. Schorb M, Haberbosch I, Hagen WJH, Schwab Y, Mastronarde DN. Software tools for automated transmission electron microscopy. *Nat Methods*. 2019;16(6):471-477.

9. Zheng SQ, Palovcak E, Armache JP, Verba KA, Cheng Y, Agard DA. MotionCor2: anisotropic correction of beam-induced motion for improved cryo-electron microscopy. *Nat Methods*. 2017;14(4):331-332.

10. Rohou A, Grigorieff N. CTFFIND4: Fast and accurate defocus estimation from electron micrographs. *J Struct Biol*. 2015;192(2):216-21.

11. Scheres SH. RELION: implementation of a Bayesian approach to cryo-EM structure determination. *J Struct Biol*. 2012;180(3):519-30.

12. Olsen RHJ, DiBerto JF, English JG, et al. TRUPATH, an open-source biosensor platform for interrogating the GPCR transducerome. *Nat Chem Biol*. 2020;16(8):841-849.

13. Fu Y, Huang Y, Yang Z, et al. Cartilage oligomeric matrix protein is an endogenous beta-arrestin-2-selective allosteric modulator of AT1 receptor counteracting vascular injury. *Cell Res*. 2021;31(7):773-790.

14. Yu J, Zhou Y, Tanaka I, Yao M. Roll: a new algorithm for the detection of protein pockets and cavities with a rolling probe sphere. *Bioinformatics*. 2010;26(1):46-52.

15. Yan C, Zou X. Predicting peptide binding sites on protein surfaces by clustering chemical interactions. *J Comput Chem*. 2015;36(1):49-61.

**Supplemental Figures**


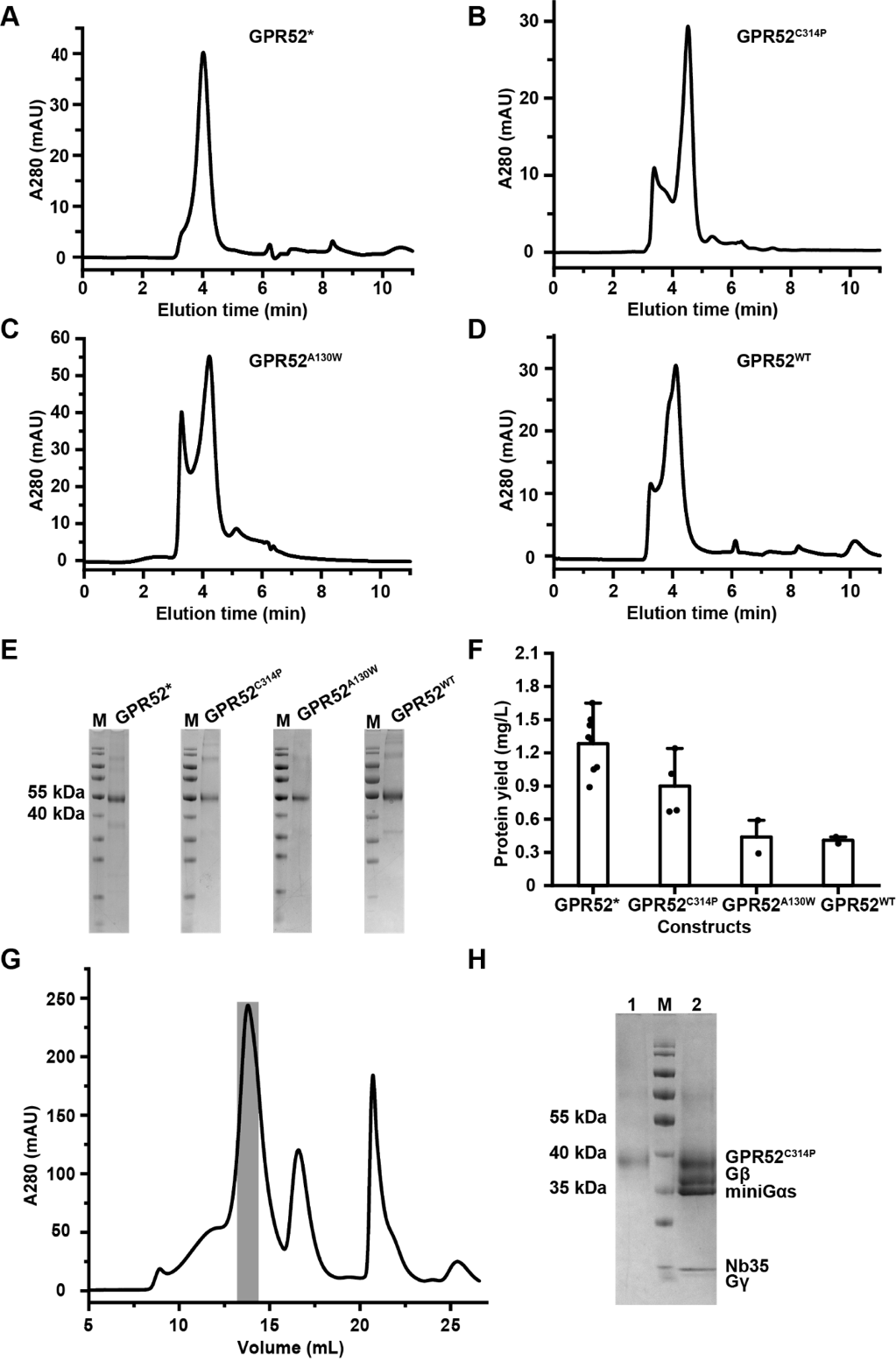


**Figure S1. Sample preparation of GPR52 for NMR study.** (A – D) Analytical size exclusion chromatography of GPR52 and its variants. (E) The SDS-PAGE results of GPR52 samples (A-D). (F) Histogram of protein yield of 1 L biomass in *Sf9* insect cell expression system. The error bar is obtained by multiple experiments. (G) Size-exclusion chromatography of GPR52^C314P^–c17–G-protein complex. The protein fractions used for NMR measurement are shown in grey square. (H)Corresponding SDS–PAGE gel of GPR52^C314P^. Lane 1: GPR52^C314P^ without N-terminal fusion protein; Lane 2: collected fraction from the grey area of (G); M: marker.


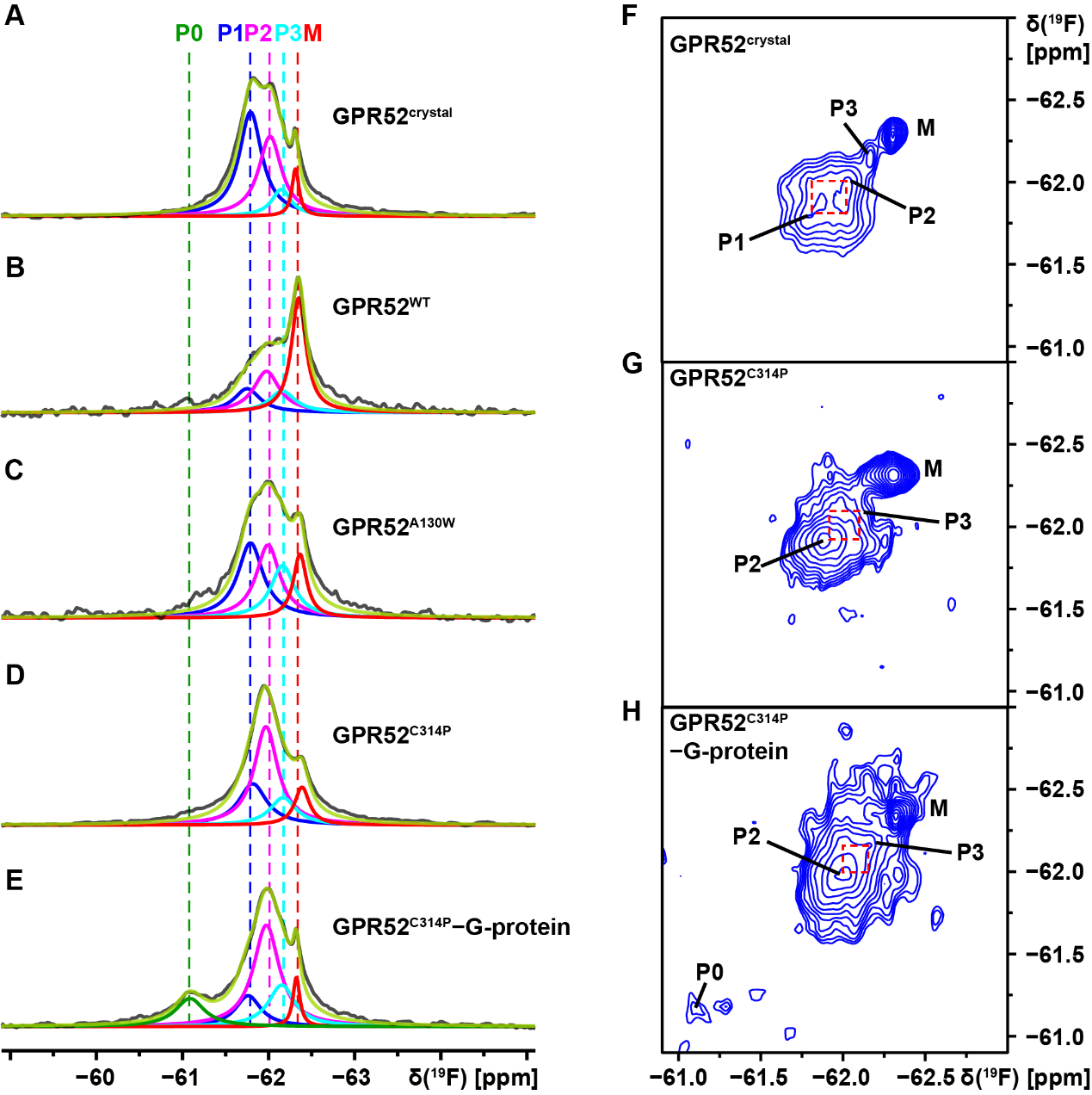


**Figure S2. Conformational polymorphism and slow exchange in GPR52 and its variants revealed by ^19^F-NMR observation of ligand c17.** (A–D) 1D ^19^F-NMR spectra of c17 in complex with different GPR52 variants in mixed micelles of LMNG/CHS at 298 K. (E) 1D ^19^F-NMR spectra of GPR52^C314P^–c17 bound to G-protein. In the NMR spectra, different individual components P0, P1, P2, P3 and M, were presented same as in Figure 1D. (F–H) Conformational exchange in the GPR52–c17 complex observed by 2D exchange spectroscopy. The samples in (F–H) are GPR52^crystal^–c17, GPR52^C314P^–c17 and GPR52^C314P^–c17–G-protein, respectively. 2D [^19^F,^19^F]-EXSY spectra were collected at 298 K with a mixing time of 400 ms. The diagonal peak positions are labeled. The red box indicates the positions of diagonal and cross peaks.


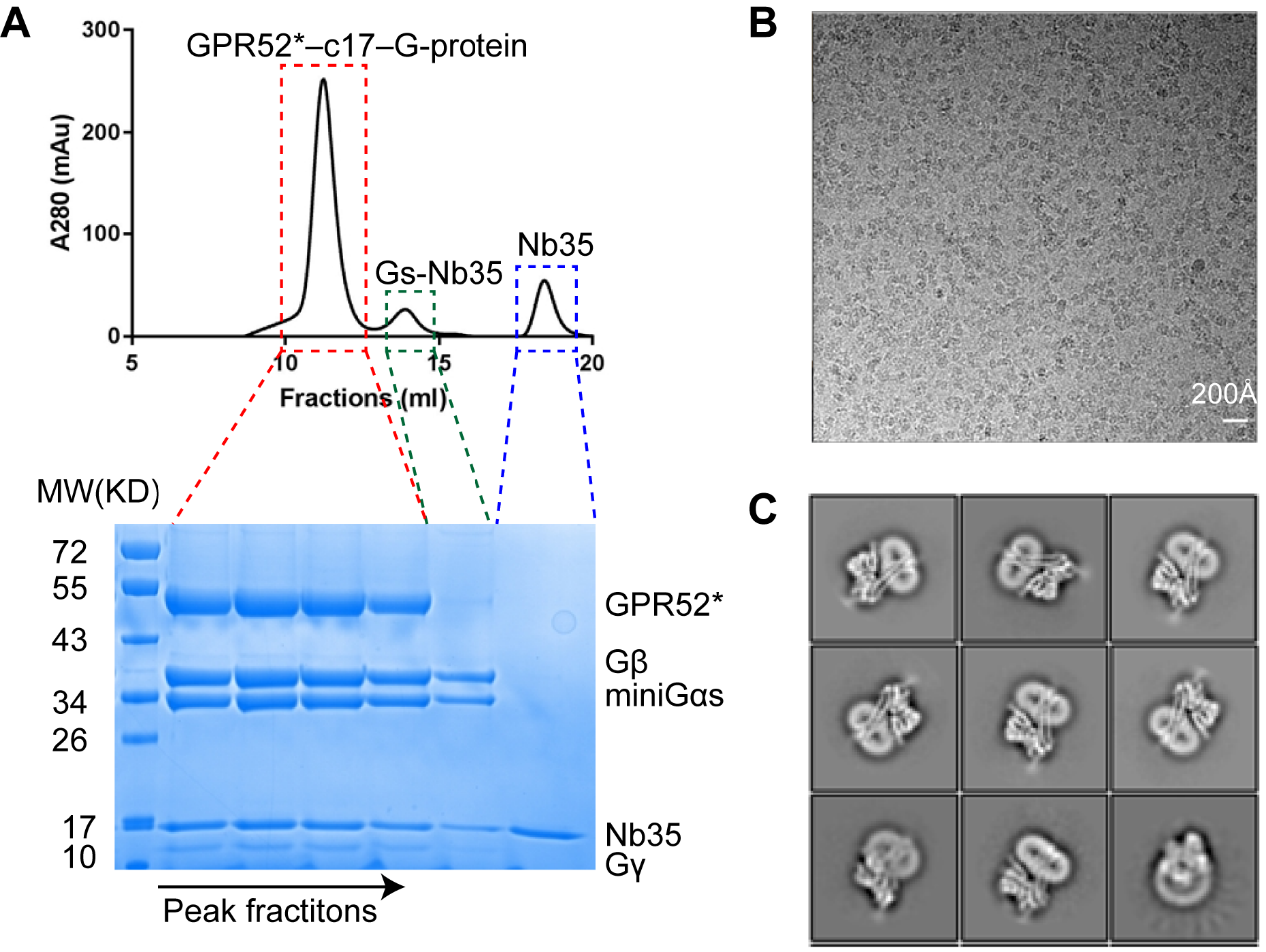


**Figure S3. Assembly of GPR52*–c17–G-protein complex for cryo-EM studies.** (A) Analytical size-exclusion chromatography (SEC) of the purified GPR52*–c17–G-protein complex, and SDS–PAGE analyses of complex after SEC. (B and C) Representative cryo-EM micrograph (B) and reference-free two-dimensional class averages (C) of the GPR52*–c17–G-protein complex.


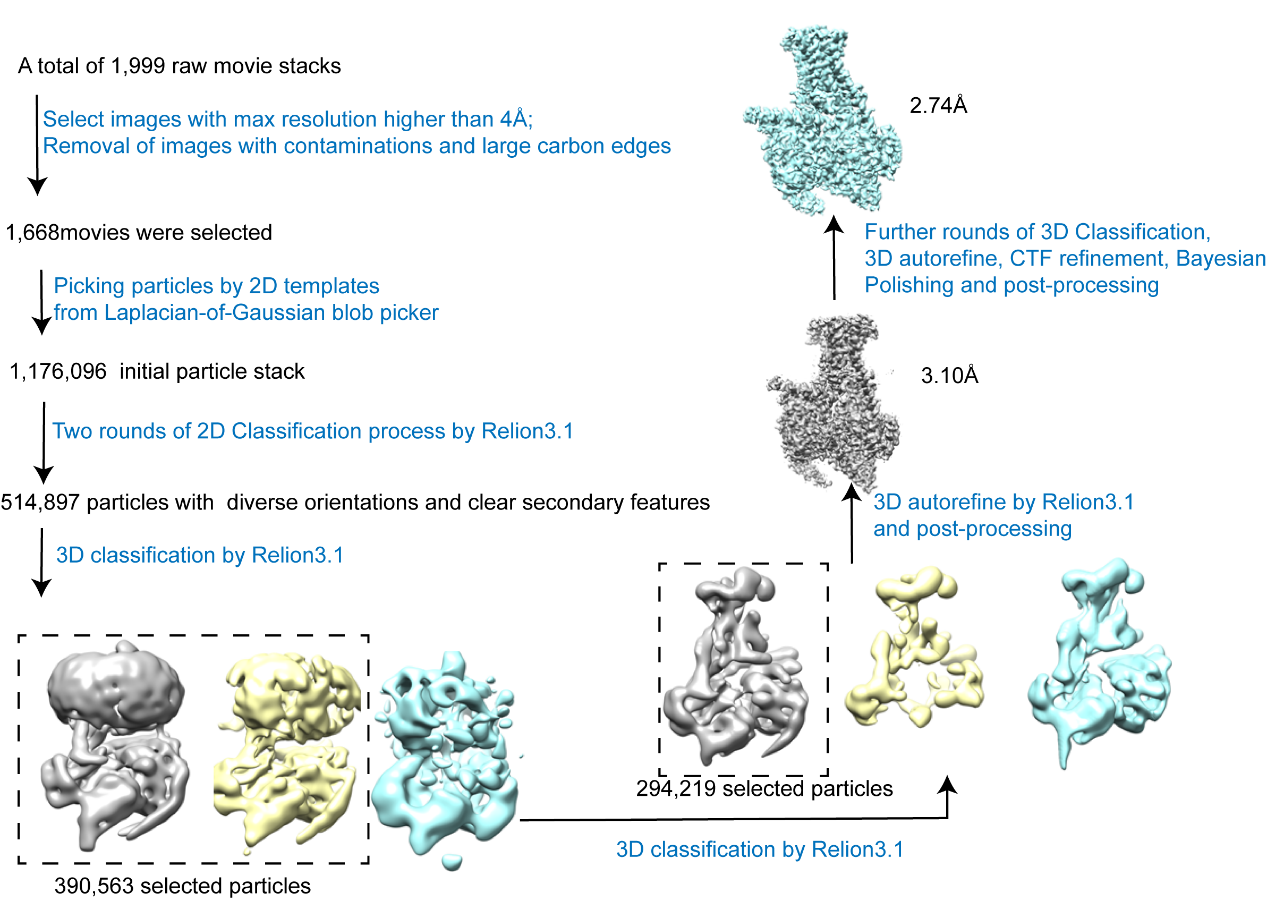


**Figure S4. Cryo-EM data processing workflow.** Workflow of cryo-EM data processing for GPR52*–c17–G-protein complex.


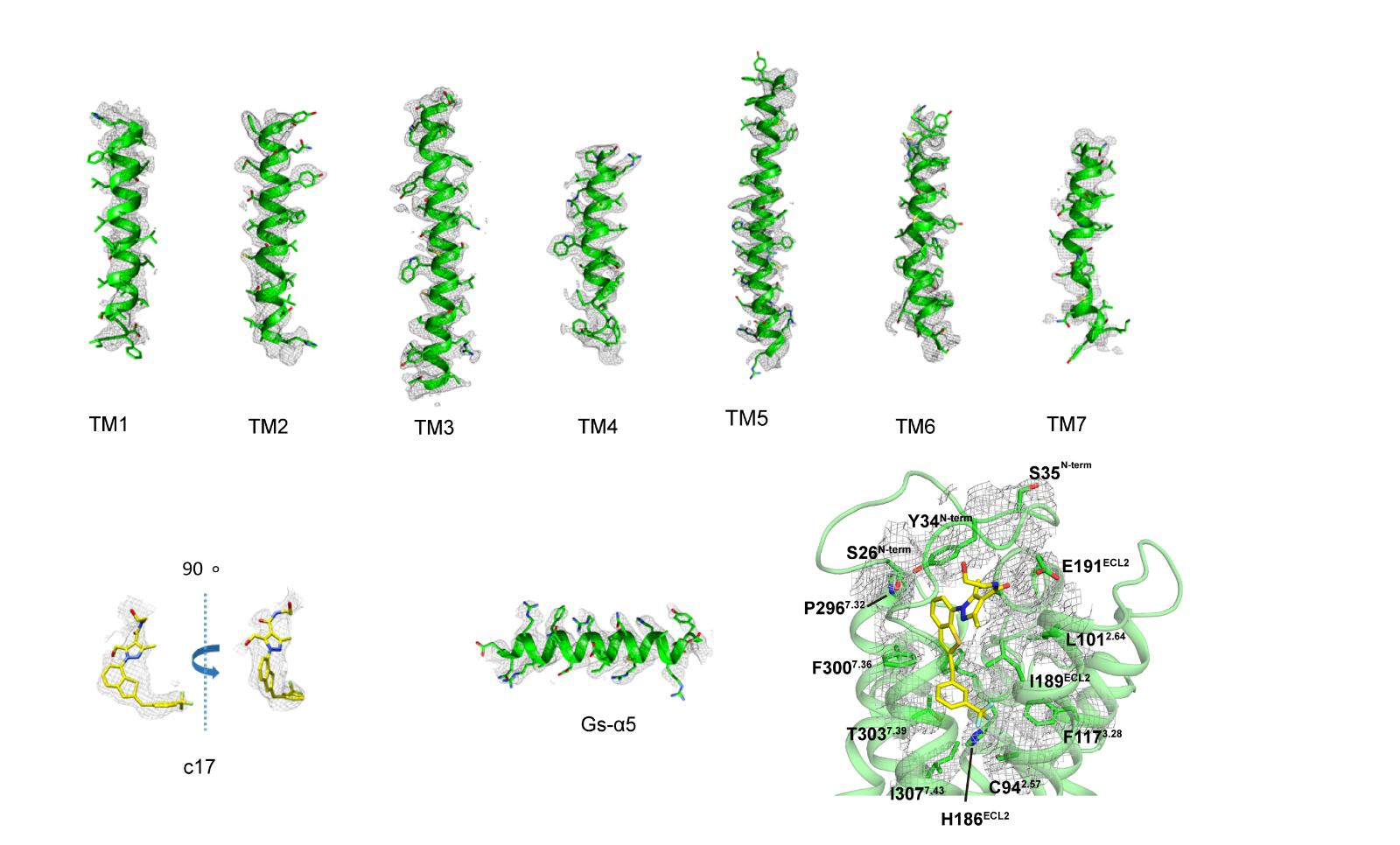


**Figure S5. Cryo-EM maps and refined structures of GPR52*–c17–G-protein complex.** Cryo-EM density map and GPR52*–c17–G-protein complex model are shown for all transmembrane helices of GPR52*, ligand c17, α5 in the miniGαs protein, and the key residues in the c17 pocket.


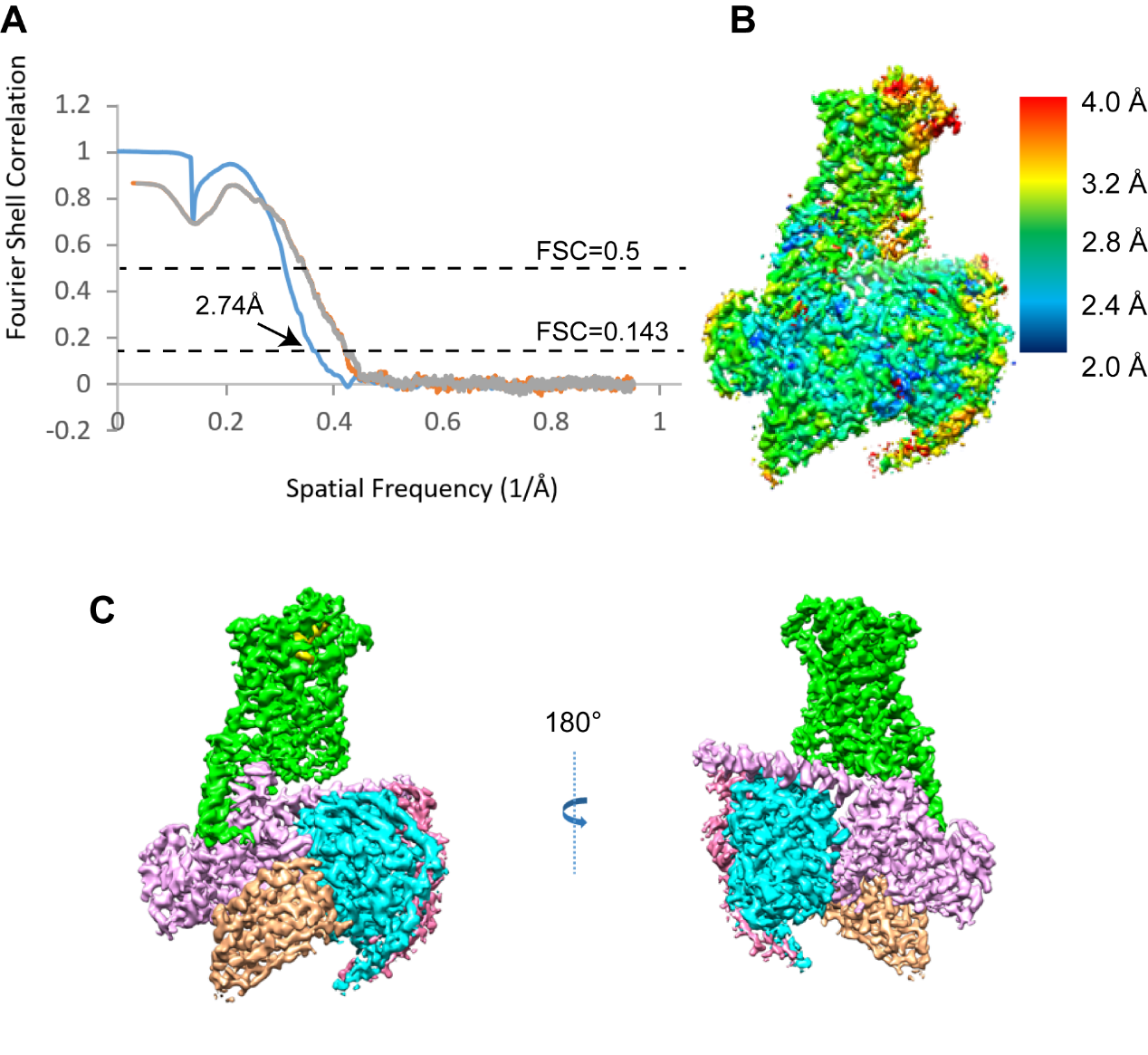


**Figure S6. Assessment of model quality.** (A) Gold standard FSC curve of the GPR52*–c17–G-protein complex. Blue curve: gold standard FSC between halfmaps; orange curve: FSC between map and model1 (GPR52*–c17–G-protein_work_); grey curve: FSC between map and model2 (GPR52*–c17–G-protein_test_). (B) Cryo-EM map to show the side view of the structure, colored according to the local-resolution estimation. (C) Cryo-EM map of GPR52*–c17–G-protein complex.


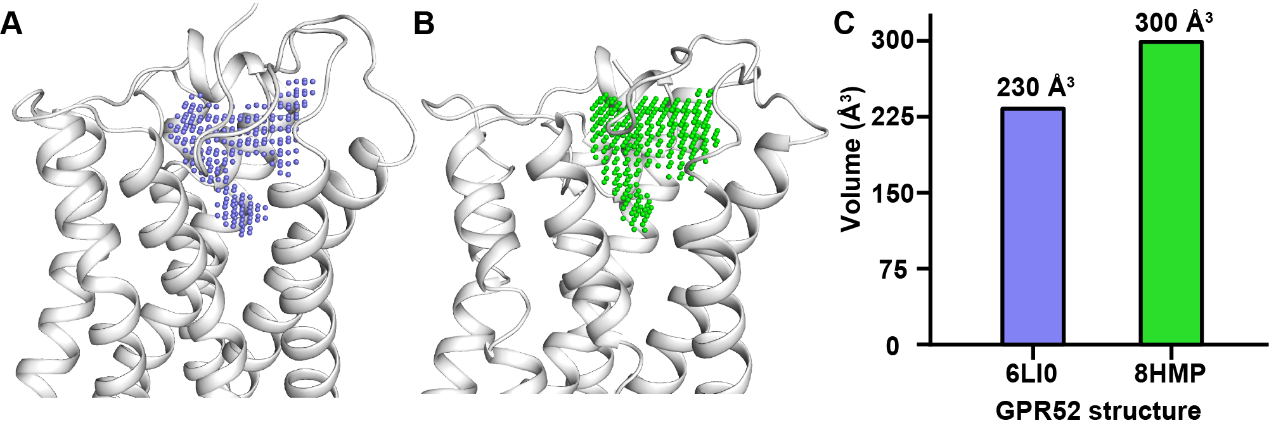


**Figure S7. The GPR52 binding pocket for ligand c17.** (A and B), The side-pocket volume in the structures of GPR52^crystal^–c17 complex (PDB: 6LI0, blue) and GPR52*–c17–G-protein complex (PDB: 8HMP, green). The pocket volumes were calculated by software POCASA and shown as spheres. (C) Histogram of ligand binding pocket volume in different GPR52 structures.


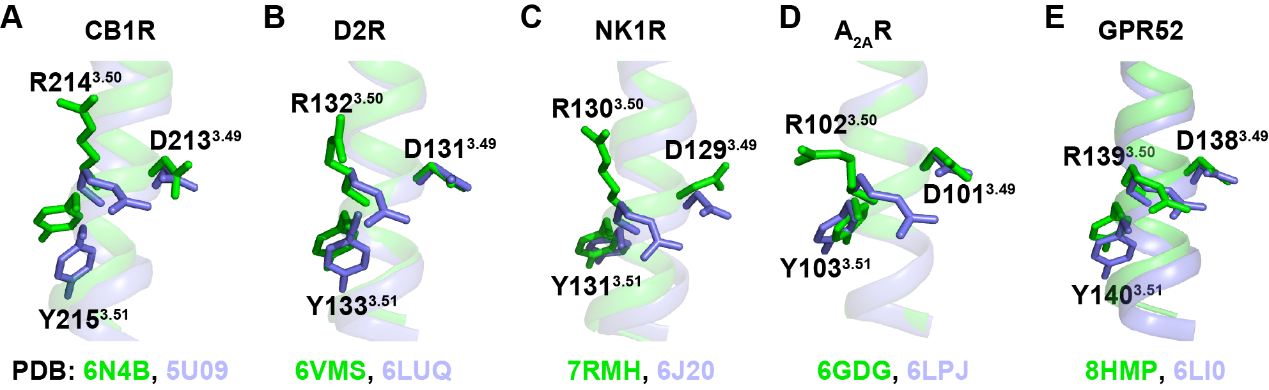


**Figure S8. The “release” of R^3.50^ in the “DRY” motif in representative active-state GPCRs.** The comparison of DRY motif in active (green) bound with G-protein and inactive (blue) structures for representative GPCRs (A–D) and for GPR52 (E). The PDB numbers of different structures were shown in the bottom.


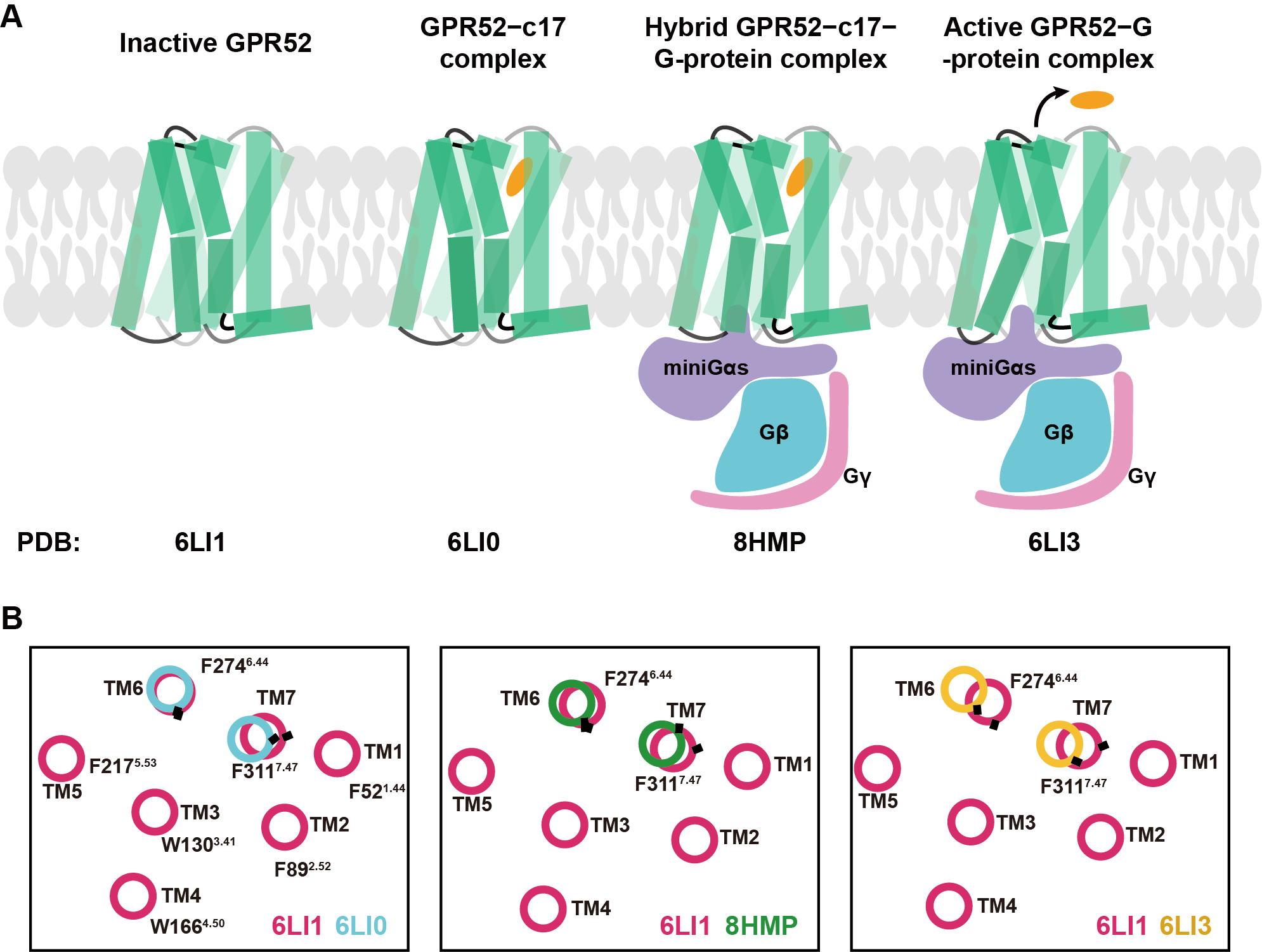


**Figure S9.** **Hypothetical activation model for GPR52.** GPR52 in the apo-form or bound with c17 cannot stabilize the receptor in an active state. The GPR52–c17–G-protein complex adopts a “hybrid” or intermediate state until c17 is released from the allosteric binding pocket. With the release of c17, the GPR52–G-protein complex retains an active state. (A) From left to right, inactive state in the absence of both c17 and G-protein (PDB: 6LI1); a stable GPR52–c17 complex in an inactive state (PDB: 6LI0); GPR52–c17–G-protein complex in a “hybrid” state (PDB: 8HMP); upon release of c17 the GPR52–G-protein complex remains in an active state (PDB: 6LI3). GPR52, green; c17, orange; Gαs, purple; Gβ, cyan; Gγ, pink. (B) The overall comparison of GPR52 structures. Transmembrane helixes are consistent based on the Cα of residues (F52^1.44^, F89^2.52^, W130^3.41^, W166^4.50^, F217^5.53^, F274^6.44^, F311^7.47^), and major differences are located in TM6 and TM7 from bottom view. The circles represent transmembrane helixes of GPR52 (6LI1, carmine; 6LI0, cyan; 8HMP, green; 6LI3, orange), and Cα of F274^6.44^ and F311^7.47^ are shown in black rectangles.

**Table S1. Construct information of GPR52**

| **Name** | **Description** (all contain BRIL and Flag tag at N-term and His tag at C-term) |
| --- | --- |
| GPR52* | GPR52[1–340, A130W, C314P] |
| GPR52^crystal^ | GPR52[1–340, A130W, A264L, W278Q, C314P, S318A, N321D, V323T], ICL3[236-261] is replaced with flavodoxin |
| GPR52^WT^ | GPR52[1–340] |
| GPR52^A130W^ | GPR52[1–340, A130W] |
| GPR52^C314P^ | GPR52[1–340, C314P] |

**Table S2.** **Ring current shift calculation of c17 in the receptor-bound state**

| PDB ID | Pseudo-atom^a^ | δ_R_ (ppm)^b^ | Contributing residue^c^ |
| --- | --- | --- | --- |
| 6LI0 | F | 0.19 | F117 |
| 8HMP | F | 0.23 | F117 |

^a^ The pseudo-atom position is at the average of the coordinates of the three fluorine atoms of the trifluoromethyl group.

^b^ Calculated using the Johnson-Bovey equation in the program MOLMOL.

^c^ Residue with largest contribution to the ring current shift.

**Table S3.** **Lorentzian deconvolution of ^19^F-NMR spectra of the GPR52*–c17 and GPR52*–c17–G-protein complexes reconstituted in LMNG/CHS micelles**

| Complex | Peak^a^ | Chemical shift (ppm)^b^ | Line width (Hz) | Integral (%) |
| --- | --- | --- | --- | --- |
| GPR52*–c17 | P1 | −61.8 | 181 | 32.8 |
|  | P2 | −62.0 | 168 | 45.3 |
|  | P3 | -62.1 | 172 | 21.9 |
| GPR52*–c17–G-protein | P0 | −61.1 | 230 | 15.0 |
|  | P1 | −61.8 | 212 | 22.1 |
|  | P2 | −62.0 | 210 | 42.0 |
|  | P3 | −62.2 | 202 | 20.9 |

^a^ See Figure 1, C and D.

^b^ ^19^F-chemical shifts were referenced to the internal standard trifluoroacetic acid at −75.5 ppm. The experiments were performed at 298 K.

**Table S4.** **Lorentzian deconvolution of ^19^F-NMR spectra of GPR52–c17 complexes reconstituted in LMNG/CHS micelles at 298 K**

| Complex | Peak^a^ | Chemical shift (ppm)^b^ | Line width (Hz) | Integral (%) |
| --- | --- | --- | --- | --- |
| GPR52^crystal^–c17 | P1 | −61.8 | 180 | 50.3 |
|  | P2 | −62.0 | 182 | 38.8 |
|  | P3 | −62.2 | 152 | 10.9 |
| GPR52^WT^–c17 | P1 | −61.8 | 205 | 27.6 |
|  | P2 | −62.0 | 205 | 47.4 |
|  | P3 | −62.2 | 205 | 25.0 |
| GPR52^A130W^–c17 | P1 | −61.8 | 200 | 39.0 |
|  | P2 | −62.0 | 188 | 35.5 |
|  | P3 | −62.2 | 188 | 25.5 |
| GPR52^C314P^–c17 | P1 | −61.8 | 220 | 27.7 |
|  | P2 | −62.0 | 185 | 55.3 |
|  | P3 | −62.2 | 205 | 17.0 |
| GPR52^C314P^–c17–G-protein­­­ | P0 | −61.1 | 229 | 15.3 |
|  | P1 | −61.8 | 200 | 14.7 |
|  | P2 | −62.0 | 205 | 49.2 |
|  | P3 | −62.2 | 210 | 20.8 |

^a^ See Supplementary information Figure S2, A–E.

^b^ ^19^F-chemical shifts were referenced using an internal standard of trifluoroacetic acid at −75.5 ppm.

**Table S5. Cryo-EM data collection, model refinement and validation statistics**

|  | GPR52*–c17–G-protein |
| --- | --- |
| **Data collection and processing** |  |
| Magnification | 105,000 |
| Voltage (kV) | 300 |
| Electron exposure (e-/ Å^2^) | 60 |
| Defocus range (µm) | -0.7 to -2.2 |
| Pixel Size (Å) | 0.832 |
| Symmetry imposed | C1 |
| Initial particle images (no.) | 1,176,096 |
| Final particle images (no.) | 294,219 |
| Map resolution (Å) | 2.7 |
| FSC threshold | 0.143 |
| Map resolution range (Å) | 1.9 ~ 5.5 |
| **Refinement** |  |
| Map sharpening B factor (Å^2^) | -14.4276 |
| Model composition |  |
| Non-hydrogen atoms | 8,319 |
| Protein residues | 1,045 |
| Ligands | EN6:1 |
| B factors (Å^2^) |  |
| protein | 62.21 |
| Ligand | 94.15 |
| R.m.s. deviations |  |
| Bond lengths (Å) | 0.005 (0) |
| Bond angles (°) | 0.895 (4) |
| Validation |  |
| MolProbity score | 1.54 |
| Clash score | 5.95 |
| Poor rotamers (%) | 0.00 |
| Ramachandran plot |  |
| Favored (%) | 96.61 |
| Allowed (%) | 3.39 |
| Disallowed (%) | 0.00 |
